# Defining fever-related discomfort in children: an international Delphi consensus

**DOI:** 10.1007/s00431-026-07340-4

**Published:** 2026-09-02

**Authors:** Gregorio P. Milani, Edward Purssell, David Martin, François Corrard, Eunicia Tan, Janice E. Sullivan, Eefje G. P. M. de Bont, Santiago Mintegi, Mario G. Bianchetti, Sebastiano A. G. Lava, Elena Chiappini

**Affiliations:** 1 Department of Pharmacy, Health and Nutritional Sciences, University of Calabria, Rende, Italy; 2 Pediatric Unit, Annunziata Hospital, Ospedale Annunziata, Via Felice Migliori, 1, 87100 Cosenza, CS Italy; 3 Little Havens Children’s Hospice, Benfleet, Essex, UK; 4 Anglia Ruskin University, Chelmsford, Essex, UK; 5 School of Medicine, Faculty of Health, Witten/Herdecke University, Witten, Germany; 6 Department of Pediatrics, University of Tübingen, Tübingen, Germany; 7 ACTIV, 31 Rue Le Corbusier, 94000 Créteil, France; 8 AFPA, 15 Rue Maurice Berteaux, 33400 Talence, France; 9 Department of Surgery, Faculty of Medical and Health Sciences, University of Auckland, Auckland, New Zealand; 10 University of Louisville School of Medicine, Louisville, USA; 11 Department of Family Medicine, CAPHRI, Maastricht University, Maastricht, The Netherlands; 12 Pediatric Emergency Department, Biobizkaia Health Research Institute Cruces University Hospital EHU, University of the Basque Country, Osakidetza, Biscay Spain; 13 Faculty of Biomedical Sciences, Family Medicine Institute, Università Della Svizzera Italiana, Lugano, Switzerland; 14 Pediatric Cardiology Unit, Department of Pediatrics, Centre Hospitalier Universitaire Vaudois, and University of Lausanne, Lausanne, Switzerland; 15 Department of Health Sciences, University of Florence, Florence, Italy; 16 Paediatric Infectious Diseases Unit, Meyer Children’s University Hospital, IRCCS, Florence, Italy; 17 Emergency Department, Middlemore Hospital, Health New Zealand Counties Manukau, Auckland, New Zealand

**Keywords:** Discomfort, Distress, Fever, Pain, Pediatric

## Abstract

Fever is one of the most common symptoms in childhood and a frequent reason for medical consultation. Current guidelines recommend treating fever only when discomfort is present. However, the concept of fever-related discomfort remains poorly defined and inconsistently applied in clinical practice and research studies. We aimed to develop an expert-based definition and a pragmatic clinical framework for the assessment and management of discomfort in febrile children. An international modified Delphi consensus was conducted to develop an operational definition and pragmatic clinical framework for fever-related discomfort in children. A steering international committee developed initial statements, which were evaluated across three iterative online Delphi rounds by seven international experts from high-income countries. Agreement was assessed using a 5-point Likert scale, with consensus predefined as ≥ 75% agreement. Experts’ feedback was used to refine statements between rounds. The panel reached consensus that fever-related discomfort is a multidimensional construct encompassing behavioral, emotional, and functional indicators. A traffic-light system was developed to stratify discomfort severity into: green (no intervention needed); amber (case-by-case intervention); and red (intervention always indicated) categories. Key clinical indicators were identified for each category. The framework was considered applicable across pediatric age groups and care settings, with individualized clinical judgment recommended for children with chronic conditions or those in intensive care.

*Conclusion*: This international Delphi consensus provides a preliminary expert-derived framework for defining and assessing fever-related discomfort in children. Future studies are warranted to evaluate its reliability, feasibility, cultural applicability, and clinical validity across larger and more diverse populations and healthcare settings.

**Table:** 

What is known: • Current pediatric guidelines recommend treating fever in children only when discomfort is present rather than aiming to normalize body temperature. • However, fever-related discomfort remains poorly defined, with substantial heterogeneity across studies and guidelines and no standardized clinical framework currently available.	
What is new: • This international Delphi consensus defines fever-related discomfort as a multidimensional construct involving behavioral, emotional, and functional indicators. • A pragmatic Traffic-Light System was developed to stratify discomfort severity and support clinical decision-making across pediatric settings and age groups.

**Supplementary Information:**

The online version contains supplementary material available at https://doi.org/10.1007/s00431-026-07340-4.

## Introduction

Fever is among the most common symptoms of illness in childhood and a major reason for pediatric consultations and caregiver concern. Although fever is a physiological host-defense mechanism, it can be associated with considerable discomfort in children [1, 2]. Historically, fever management has focused on lowering body temperature; however, there is a consensus across current guidance emphasizing that relief of discomfort or pain should be the primary therapeutic goal [1, 2].

Despite this paradigm shift, inappropriate practices in fever management persist among caregivers and healthcare professionals, and the phenomenon described as “fever phobia,” which refers to the undue anxiety of fever itself, continues to influence clinical decision-making and parental anxiety [3, 4]. The persistent focus on temperature reduction rather than on the child’s clinical condition may lead to unnecessary or inappropriate therapeutic interventions, including excessive use of antipyretics, physical cooling methods or even antibiotics [5–7]. On the other hand, previous studies have shown discomfort might be independent of the height of fever, suggesting that partially different metabolic pathways underlie these conditions [8, 9].

Previous observational and interventional studies considered discomfort as a broad range of manifestations, including pain, irritability, crying, chills, and behavioral changes, rather than being limited to body temperature or pain alone [10, 11]. However, the concept of discomfort remains inconsistently defined, with substantial heterogeneity in its definition, management, and clinical interpretation across studies and guidelines, and no universally accepted standardized definition is currently available [10, 12].

Given that most current pediatric guidelines recommend administering antipyretics only when discomfort is present, the absence of a shared definition and assessment framework represents a major gap in pediatric fever management and may hinder evidence-based clinical practice [12]. Therefore, improving the conceptualization and clinical operationalization of fever-associated discomfort is essential to optimize therapeutic decisions, reduce unnecessary treatments, and align clinical practice with guideline recommendations.

To address this gap, we conducted an international modified Delphi consensus to develop an expert-based definition and a pragmatic clinical framework for the assessment and management of discomfort in febrile children.

## Material and method

### Study design

A modified Delphi approach was used to reach expert consensus on the definition of discomfort in febrile children. The modified Delphi is a variation of the traditional Delphi technique that combines qualitative feedback with quantitative ratings across repeated rounds [13]. In this approach, participants review a structured set of items developed from literature research. This method was chosen to ensure participant anonymity and to facilitate a structured, iterative evaluation process. The process adhered to the ACCORD recommendations for reporting consensus methodology [14]. The study procedures were defined and written a priori before the study onset.

### Steering committee and literature review

The process was led by a four-member steering committee (two from Italy—G.P.M. and E.C., and two from Switzerland—S.A.G.L. and M.G.B.), which conducted the literature review and drafted the initial statements. The draft statements were based on a systematic review previously conducted by some members of the committee [10]. Briefly, PubMed, Embase, and Cochrane databases were searched from inception to November 30, 2023, using terms related to children, fever, and discomfort. National and international guideline repositories were also examined. Eligible studies included original publications in English that defined discomfort in febrile children. Fourteen studies provided a definition of discomfort. The provided definitions, which varied widely, including crying, irritability, chills, pain, distress, or facial changes, were used as a basis to develop an initial structured definition of discomfort.

### Participants

Seven physicians with extensive experience managing pediatric fever were selected and invited in September 2024 by the steering committee. All had at least 15 years of documented experience, peer-reviewed publications in the field or prior participation in at least one national or international consensus on the management of febrile children. Panelists were recruited from high-income countries (France—F.C., Germany—D.M, New Zealand—E.T., Spain—S.M., the Netherlands—E.D., the United Kingdom—E.P., the United States of America—J.E.S.) to ensure a broad international perspective applicable across diverse high-income settings. All these panel members were invited directly by the steering committee via email (no more than two invitations were sent) and agreed to participate. No reimbursement or other type of incentive was provided.

### Questionnaire

There were 20 initial items identified from the literature (Supplementary online material), covering domains relevant to the definition and management of febrile discomfort. The first group of items (#1–2) addressed the conceptual framework, asking panelists whether discomfort should be considered a multidimensional construct and whether the presence of one or more indicators was sufficient to guide clinical decisions. For the specific task of ranking the most relevant child characteristics for defining discomfort, a traffic-light system (green = no intervention needed; amber = case-by-case intervention; and red = intervention always advised) was introduced after the first round of voting.

A second group of items examined specific clinical indicators, including reduced hydration or food intake (#3a), diffuse myalgia or arthralgia (#3b), chills or piloerection (#3c), self-reported malaise (#3d), prolonged or inconsolable crying (#3e), nonverbal signals such as moaning or a distressed facial expression (#3f), irritability or restlessness (#3 g), marked fatigue or decreased responsiveness (#3 h), and sleep disturbances (#3i). Additional items clarified the definition’s boundaries by specifying exclusion criteria: localized pain (to be assessed with validated pain scales) (#4) and overall well-being (measurable with validated tools but not equivalent to discomfort) (#5). Further items addressed applicability across pediatric age groups (#6), comorbidities (#7), and care contexts, including hospital, outpatient, home, or school settings (#8), as well as potential use by different healthcare professionals (#9–10).

The final items addressed aspects of management and follow-up, specifically treatment rather than prevention (#11) and the need for reassessment after an appropriate interval (#12).

### Delphi procedure

Once the preliminary items were determined, they were pilot tested by the four members of the steering committee to ensure clarity and appropriateness before being shared with the expert panel. To reduce bias, committee members abstained from voting. Between March and July 2025, three iterative online rounds were conducted. In each round, panelists rated their agreement with each item on a 5-point Likert scale (“strongly agree” to “strongly disagree”). Responses of “strongly disagree,” “disagree,” or “neither agree nor disagree” (scores 1–3) required a free-text comment, which was analysed and used to revise items for subsequent rounds. In later rounds, only items that had not met the predefined consensus threshold were re-circulated for voting.

### Analysis and consensus definition

Responses were collected electronically through Microsoft Forms and analysed anonymously. After each round, participants received summarized feedback, including agreement percentages, medians, and interquartile ranges, to guide their re-evaluation. Consensus was a priori defined as ≥ 75% of panellists rating “agree” or “strongly agree.” Based on the comments, items that did not reach the consensus were modified by the steering committee before re-evaluation by the panellists. The mean rate of agreement was also calculated for each item as follows: “strongly disagree” = 1, “disagree” = 2, “neither agree nor disagree” = 3, “agree” = 4, and “strongly agree” = 5.

## Results

All panel experts completed the three Delphi rounds. In round 1, consensus was achieved for 7 of 20 statements. In round 2, seven statements were reassessed, including introduction of the Traffic Light System, which incorporated 9 statements, seven of which had not previously reached consensus. Three statements required a third round to reach ≥ 75% agreement, and one statement did not reach agreement.

Experts agreed that fever-associated discomfort cannot be defined by a single sign or symptom but rather by multiple clinical indicators that may occur concurrently or independently (100% agreement).

**Fig. 1:**
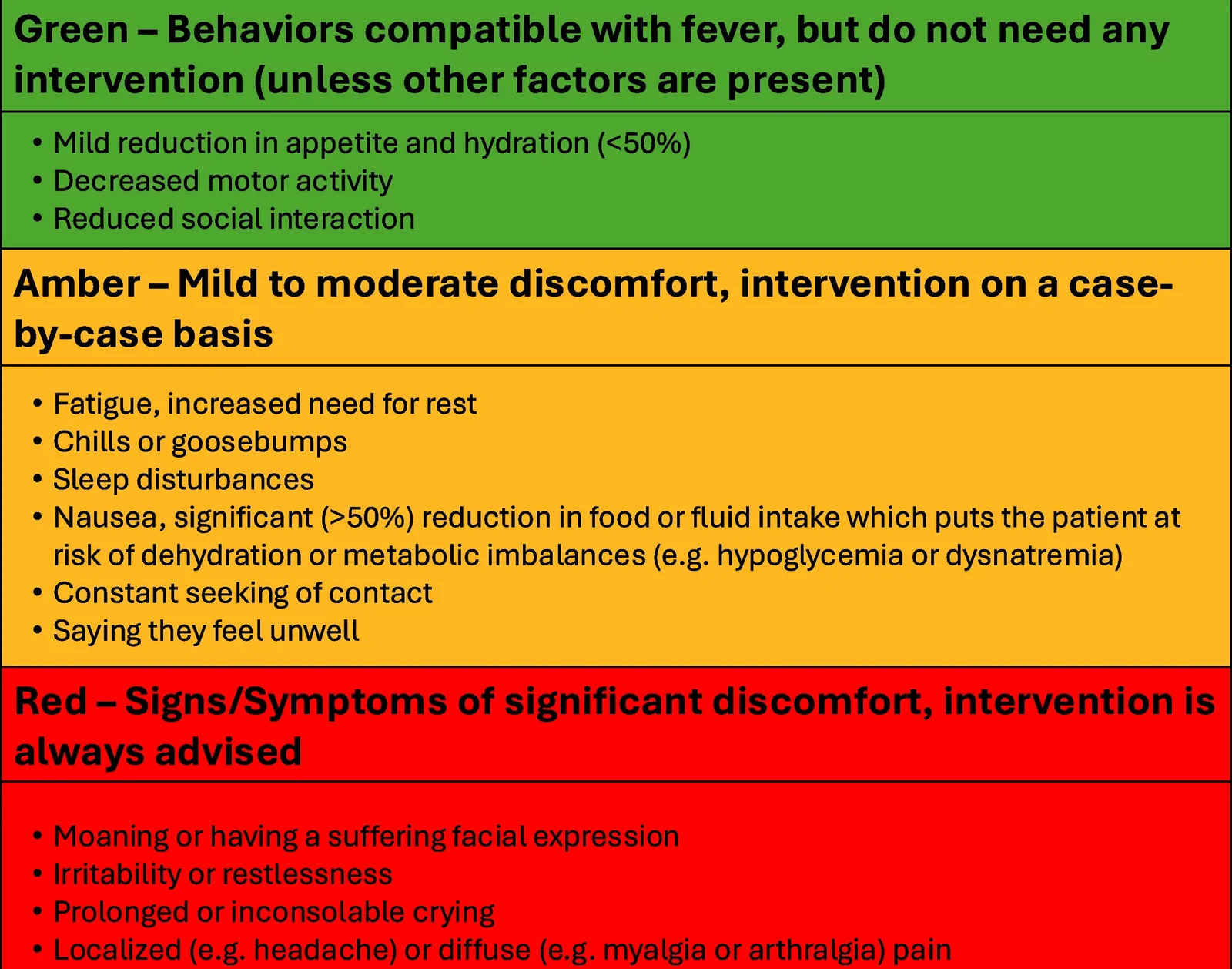
Traffic light system for discomfort approach

In round 1, experts assessed potential indicators of fever-associated discomfort. Moaning or a suffering facial expression reached the highest agreement (86%), whereas reduced hydration or caloric intake, chills or goosebumps, and fatigue or reduced responsiveness reached the lowest agreement (57%). Given this variability, the steering committee introduced a Traffic Light System in round 2 to classify indicators as green (no treatment required), amber (treatment considered case by case), or red (treatment required). After revision based on panel feedback, full consensus was achieved in round 3 on both the indicators included and their stratification. All experts agreed to adopt the Traffic Light System (Fig. 1) for identifying and managing fever-related discomfort, and 86% agreed that the presence of at least one amber or red indicator suggests potentially significant discomfort. Experts also agreed that “well-being” should not be considered synonymous with comfort/discomfort (86%). Pain was considered a component of discomfort but not equivalent to it; localized pain should be assessed using validated pain scales, although it may also represent a sign of fever-associated discomfort (100%). The definition of discomfort was considered applicable across the pediatric age spectrum (86%), although experts recommended case-by-case application in children with significant comorbidities or in intensive care settings (86%). The panel also agreed that the definition can be used by nurses and other health care professionals and across hospital, outpatient, and community settings (both 86%).

The panel also evaluated the definition of fever. The World Health Organization definition (axillary temperature > 37.5 °C) was not approved by any expert. In subsequent rounds, 71% agreed that no single temperature threshold, measurement site, or universal definition should be applied when assessing fever-related discomfort, and that definitions vary across the literature and according to national or local guidelines. However, no consensus on the definition of fever was reached.

Finally, 86% of experts agreed that management should focus on therapeutic rather than preventive interventions and that the child’s condition should be reassessed after treatment within a reasonable timeframe.

Results of the three-round Delphi process are listed in Table 1.

**Table 1:** Results of the three-round Delphi process on the definition and assessment of fever-associated discomfort in children

First round statements	Mean	Agreement	Second round statements	Mean	Agreement	Third round statements	Mean	Agreement
**1-**Fever-associated discomfort cannot be defined as a single sign or symptom; rather, it comprises multiple clinical indicators that may present concurrently or independently	4.86	100%						
			**Traffic Light System for Discomfort Symptoms in Febrile Children (**Fig. 1**)** This colour-coded classification guides clinical decision-making by grouping behavioural symptoms associated with fever into three categories: • **Green:** Behaviors compatible with fever that generally do not require intervention • **Amber:** Signs of mild to moderate discomfort requiring clinical judgment • **Red:** Indicators of significant discomfort where intervention is always advised	3.71	57.1%	**Traffic Light System for Discomfort Symptoms in Febrile Children (**Fig. 1**)** This colour-coded classification guides clinical decision-making by grouping behavioural symptoms associated with fever into three categories: • **Green: Behaviors compatible with fever, but do not need any intervention (unless other factors are present)** • **Amber: Mild to moderate discomfort, intervention on a case-by-case basis** • **Red: Signs/Symptoms of significant discomfort, intervention is always advised**	4.43	100%
**2-**If at least one of the indicators of discomfort is present in a child with fever, the presence of discomfort should be diagnosed and managed accordingly	3.86	71.4%	If at least one of the indicators of discomfort is present in a child with fever, the presence of discomfort should be considered	3.86	57.1%	If at least one of the indicators (amber and red signs/symptoms in the traffic light system) is present in a child with fever, the presence of a potentially significant discomfort should be considered	4.14	85.7%
3a-A reduced ability to maintain adequate hydration and caloric intake during the past 8 h is a possible indicator of fever-associated discomfort	3.14	57.1%	Included in Traffic Light System Statement					
3b-Diffuse myalgia or arthralgia are possible indicators of fever-associated discomfort	4.0	71.4%	Included in Traffic Light System Statement					
3c-Chills or goosebumps are possible indicators of fever-associated discomfort	3.57	57.1%	Included in Traffic Light System Statement					
3d-The child reports feeling unwell is a possible indicator of fever-associated discomfort	4.0	71.4%	Included in Traffic Light System Statement					
3e-Prolonged or inconsolable crying are possible indicators of fever-associated discomfort	4.14	71.4%	Included in Traffic Light System Statement					
3f-Moaning, or having a suffering expression are possible indicators of fever-associated discomfort	4.57	85.7%	Included in Traffic Light System Statement					
3 g-Irritability or restlessness are possible indicators of fever-associated discomfort	4.14	71.4%	Included in Traffic Light System Statement					
3 h-Fatigue or reduced responsiveness (e.g., does not get out of bed/sofa) are possible indicators of fever-associated discomfort	3.43	57.1%	Included in Traffic Light System Statement					
3i-Difficulty falling asleep, frequent awakenings, or waking earlier than usual are possible indicators of fever-associated discomfort	4.00	71.4%	Included in Traffic Light System Statement					
4-A child experiencing localized pain (even in multiple locations), for example, otalgia and/or pharyngodynia, is evaluated and treated according to existing validated pain scales; therefore, this type of condition should not be considered a symptom/sign of discomfort	4.0	85.7%	A child experiencing localized pain (even in multiple locations), for example, otalgia and/or pharyngodynia, is evaluated and treated according to existing validated pain scales	4.57	100%			
5-The evaluation of the child's “well-being” is conducted using existing validated well-being scales, and this aspect should therefore not be considered a synonym of comfort/discomfort	4.00	71.4%	The evaluation of the child's “well-being” should not be considered a synonym of comfort/discomfort	4.29	85.7%			
6-The definition of discomfort applies to the pediatric population (patients aged from one month to 17 years)	3.71	71.4%	The definition of discomfort applies to the pediatric population (considering preverbal children, children 4 to 8 years and children older than 8 up to 17 years)	4.14	85.7%			
7-The definition of discomfort applies to febrile children, where fever is defined according to the World Health Organization as an axillary temperature > 37.5 °C measured with an electronic axillary thermometer, tympanic thermometer, or other methods, but not rectal or oral	1.71	0%	Definition of fever in the context of fever associated discomfort should not be set a priori but it depends on national guidelines or local protocols	3.86	71.4%	The definition of fever varies in the literature and depends on national guidelines or local protocols	3.86	71.4%
8-The proposed definition of discomfort and subsequent antipyretic treatment, if indicated, does not apply to the following patients, for whom fever management may follow different principles (each exclusion criterion should be assessed with a Likert scale): • Children with neurological conditions/epilepsy/febrile seizures, • Children with severe heart diseases, • Children with severe metabolic disorders, • Children admitted to intensive care units	3.0	42.9%	The proposed definition of discomfort and subsequent antipyretic treatment, may follow different principles for the following groups of patients and should be considered case-by-case: • Children with neurological conditions • Children with severe heart diseases • Children with severe metabolic disorders • Children admitted to intensive care units	3.86	71.4%	The proposed definition of discomfort and subsequent therapeutic approach may follow different principles for some patients with specific conditions. The applicability of the definition and approach should be considered case-by-case for the following patients: • Children with neurological conditions • Children with severe heart diseases • Children with immunological conditions or being treated with immunosuppressive medications • Children with severe metabolic disorders • Children with severe lung diseases (bronchopulmonary dysplasia) • Children with homozygous sickle cell disease • Children admitted to intensive care units	4.29	85.7%
9-The definition of discomfort in febrile children and its assessment can be applied by physicians (both specialists and non-specialists), nurses, and other healthcare professionals involved in the routine clinical care of children	4.57	85.7%						
10-The definition of discomfort in febrile children and its assessment is applicable in the following settings: • Outside the hospital (e.g. home or school), • Outpatient clinics, • Inpatient care	4.57	85.7%						
11-Management of febrile children with discomfort involves interventions for therapeutic purposes only, not for prevention	4.57	85.7%						
12-After administering therapy for discomfort, it is appropriate to reassess the discomfort after a reasonable amount of time	4.57	85.7%						

## Discussion

Fever and fever-related discomfort remain high-impact clinical issues in pediatrics, yet their management is poorly standardized and often clinically inconsistent. International and national guidelines, including those from the Italian Pediatric Society, National Institute for Health and Clinical Excellence (NICE), American Academy of Pediatrics (AAP), and World Health Organization (WHO), emphasize that the primary goal of fever management is the relief of discomfort rather than temperature normalization [2, 15–17]. In this context, our Delphi consensus is the first attempt to move beyond the generic principle of “treat discomfort” toward a clinically usable definition and shared framework. The results conceptualize fever-related discomfort as a multidimensional construct spanning behavioral, emotional, and functional domains that no single clinical sign can fully capture.

A key innovation of this Delphi process is a traffic-light system to stratify the severity of fever-related discomfort and guide clinical decisions. This approach has been used to produce guidance for the management of fever (NICE); therefore, the concept might be familiar to many healthcare professionals. The green category includes mild changes commonly associated with fever, such as slightly reduced appetite or hydration, decreased activity, or diminished social interaction. The main contribution of this category is to explicitly clarify that, when present in isolation, these manifestations may represent physiological correlates of fever rather than clinically significant discomfort requiring an intervention. This distinction might be essential to prevent the automatic use of antipyretics in response to any deviation from baseline behavior. Indeed, previous literature has warned against overtreatment and proposed a practical approach centered on monitoring changes in daily habits (sleep–wake rhythm, appetite, motor activity, mood, daily routines, and facial expression) to support appropriate clinical decisions and caregiver communication [18]. Despite this, surveys among pediatricians indicate that guideline-concordant behaviors remain incomplete, with persistent use of physical methods, alternating antipyretics, and only partial adoption of discomfort-driven treatment strategies [19, 20]. The green category aims therefore to counteract the “treat-the-temperature” reflex. The amber category captures mild-to-moderate discomfort, for which treatment should be considered on a case-by-case basis rather than applied automatically. Overall, the amber category encourages clinicians to interpret signs within the broader clinical context, balancing symptom relief with the avoidance of unnecessary medication. On the other hand, the red category includes symptoms or signs which deserve a prompt treatment in all cases. This approach should prevent or resolve potentially detrimental consequences of some symptoms associated with significant discomfort in children with fever. However, as highlighted in the comments by panelists, the specific intervention was not addressed in the process. Moreover, the intervention should not be intended exclusively as a pharmacological treatment: increasing data suggest that nonpharmacological approaches can significantly reduce distress in children [21]. “Feeling unwell” is included among the amber signs and symptoms, since its characteristics and associated burden should be further explored by a healthcare professional together with the patient and/or their caregivers. However, the experts emphasized a conceptual distinction between more general pediatric well-being and fever-associated discomfort. Indeed, well-being is a multidimensional construct encompassing psychological, emotional, social, and functional domains [22, 23], whereas fever-associated discomfort is an acute, transient clinical condition. The Delphi panel confirmed that these constructs should not be considered interchangeable, supporting a symptom-centered approach when evaluating febrile children.

Localized pain was considered a red sign. The experts agreed that localized pain should be systematically assessed and managed using validated pain scales [24]. At the same time, pain was not excluded from the framework of fever-associated discomfort, emphasizing that discomfort may coexist with specific pain syndromes and should be interpreted within the broader clinical context. Importantly, this new definition highlights that discomfort may be present in children with fever even if pain is absent. Only a few previous studies used structured scoring systems to assess “discomfort” or “comfort.” One trial evaluated “comfort” using a composite approach that included the CHEOPS pain scale, along with general behavior and relief ratings [25]. Another study employed the Faces Pain Scale–Revised [9]. Compared with these tools, our Traffic-Light System is not centered solely on pain assessment, but is broader and more comprehensive.

Consistent with current guidelines, the panel acknowledged that this framework cannot be applied uniformly to all children. According to the literature, chronic conditions, as well as intensive care settings, may alter baseline behavior and symptom expression, requiring individualized clinical judgment [26, 27]. Nevertheless, the panel supports the broad applicability of this strategy across different settings and professional roles. By promoting a common understanding of fever-related discomfort, this approach may enhance communication among clinicians, caregivers, and other healthcare professionals and help reduce overtreatment driven by fever phobia. In this perspective, future studies should evaluate the results of this Delphi study.

Of note, the only statement that did not meet the predefined agreement threshold of 75% concerned the definition of fever. Rather than representing a limitation of the Delphi process, this lack of consensus likely reflects the substantial heterogeneity that exists across international guidelines. Definitions of fever vary considerably with respect to temperature thresholds, measurement sites, and underlying conceptual approaches. While some guidelines use ≥ 38.0 °C as a reference cut-off, individual-, device-, age-, and site-specific thresholds are inconsistently applied, and some recommendations adopt pragmatic or risk-based approaches rather than relying on a single temperature definition [2, 15–17]. For instance, the AAP guidelines recommend rectal temperature measurement for infants and consider a fever a temperature at 38 °C or higher. On the other hand, NICE guidelines discourage the rectal route in patients between 0 and 5 years of age and provide temperature cut-off to identify patients at higher risk for serious illness instead of a cut-off for fever [15, 16]. At the end of the Delphi process, the experts agreed to proceed with a future study to develop an international consensus document to standardize fever definition in pediatric practice.

## Limitation

The Delphi panel included a limited number of experts, and all were paediatric professionals with extensive clinical and research experience in pediatric fever management. There is no universally accepted standard for the sample size of a Delphi panel. Proposed ranges vary from 5 experts [28, 29] to 30 experts [30], but several authors emphasize that the characteristics and representativeness of panellists are more important than their exact number [31]. However, the low number of experts may limit the generalizability of the findings. The Delphi methodology relies on expert opinion rather than empirical clinical data. Although this approach is appropriate for areas with limited evidence and high conceptual heterogeneity, the proposed framework requires validation in real-world clinical settings. In particular, future prospective studies are needed to assess the reliability, feasibility, and clinical impact of the proposed Traffic-Light System across different healthcare environments. Also, the evaluation of this system should be performed by different types of healthcare providers including primary care pediatricians, general physicians, nurses and caregivers. Moreover, the consensus was developed primarily for high-resource settings, and its applicability to low-resource settings, where healthcare availability, cultural perceptions of fever, and caregiver behaviors may differ, remains uncertain. Finally, and importantly, the definition of fever-related discomfort proposed in this study does not replace clinical judgment in the management of conditions associated with fever.

This international consensus developed a clinically oriented framework to define fever-related discomfort in children. By defining discomfort as a multidimensional construct and introducing a practical Traffic-Light System to grade symptom severity, it moves beyond the traditional temperature-focused approach to fever management and offers a tool to support clinical decisions. The consensus distinguishes fever-related discomfort from pain and overall well-being, promoting a symptom-centered, patient-focused approach consistent with current guidelines. Despite preliminary, the results of this study might improve communication between caregivers and healthcare professionals, helping to reduce inappropriate antipyretic use and fever phobia. Future studies are warranted to evaluate its reliability, feasibility, cultural applicability, and clinical validity across larger and more diverse populations and healthcare settings.

## FSUPPSupplementary Information

Below is the link to the electronic supplementary material.

ESM 1 DOCX (23.4 KB)

## Data Availability

All data supporting the findings of this study are available within the paper and its Supplementary Information.

## Abbreviations

NICE: National Institute for Health and Clinical Excellence
AAP: American Academy of Pediatrics
WHO: World Health Organization
