# Supplementary material for: Defining fever-related discomfort in children: an international Delphi consensus
Source: Eur J Pediatr. 2026 Sep 2;185(9):707. doi: 10.1007/s00431-026-07340-4 (PMC13534207; doi:10.1007/s00431-026-07340-4)
Supplement: Supplementary file 1 [file 431_2026_7340_MOESM1_ESM.docx]

**Supplementary material**

**Supplementary material Table S1**. Initial statements and supporting rationale underlying the Delphi consensus on fever-associated discomfort in children

| **Statements** | **Rationale** |
| --- | --- |
| **1** | Fever-associated discomfort may present with several signs and symptoms [1]. |
| **2** | Different clinical indicators that collectively address the discomfort experienced by a febrile child may be considered. Each indicator was associated with discomfort in previous studies [2; 3] . Thus, it warrants considering a child with a fever who presents one of these indicators as experiencing discomfort. |
| **3a** | Fever is frequently associated with metabolic alterations that further enhance appetite reduction. These changes are compounded by behavioral and psychological effects, such as a general sense of malaise and nausea, which further suppress appetite [3]. |
| **3b** | Muscle or joint pain commonly accompanies febrile illnesses as part of the systemic inflammatory response Children can exhibit reluctance to move or play, underscoring the need for adequate discomfort management [5]. |
| **3c** | Chills and “goosebumps” signal an active thermoregulatory mechanism, involving peripheral vasoconstriction and a subjective sense of cold [6]. This phenomenon can be distressing for the child [2]. |
| **3 d** | Self-reported being unwell is also a subjective experience that should be always considered similarly to pain [7]. |
| **3 e** | Persistent crying that exceeds normal consolation attempts reflects high distress in younger children, who lack refined verbal expressions, when presenting fever and discomfort [8]. |
| **3f** | Non-verbal signals, such as moaning or a clearly pained facial expression, point out a discomfort condition in young children, or in children otherwise unable to articulate their discomfort [8]. |
| **3g** | Irritability can stem from both the physiological impact of fever and the psychological burden of feeling unwell. Restlessness may also emerge as a proxy for overall febrile discomfort [9]. |
| **3h** | Marked tiredness or unwillingness to engage in normal activities typifies the “sickness behaviour” response to infection that may be present in patients with fever [9; 10]. |
| **3i** | Sleep disturbances, whether in onset, continuity, frequency or early awakening, are observed in febrile children with discomfort and alteration of normal circadian rhythms [4]. |
| **4** | Localized pain is typically evaluated using validated pain scales rather than being categorized as a general sign of discomfort. Pain-specific scales and well-established protocols exist to manage localized pain [11]. |
| **5** | Pediatric well-being is assessed using validated well-being scales (e.g. Yale score, Pediatric Assessment Triangle) that consider physical and behavioral aspects and are useful especially for the initial diagnostic evaluation and therapeutic approach to the potential underlying cause of the disease [12; 13]. |
| **6** | guidelines on fever associated discomfort typically refer to pediatric age [14]. |
| **7** | The World Health Organization (WHO) defines fever as an axillary temperature >37.5°C. The measurement should be performed by electronic axillary, tympanic, or other validated thermometers, but rectal and oral methods should be excluded due to measurement variability and potential patient discomfort [15]. |
| **8** | Certain pediatric populations require special fever management approaches due to their underlying conditions. In some cases, fever itself can have detrimental neurological, cardiovascular and metabolic consequences [16]. Thus, the proposed definition and approach to fever-associated discomfort should not be applied to these groups. |
| **9** | A standardized discomfort assessment should be applicable across various healthcare professionals, including physicians (both specialists and generalists), nurses, and allied healthcare providers [1]. |
| **10** | Pediatric discomfort associated to fever is not limited to hospital settings. It is relevant in home care, outpatient clinics, schools, and inpatient units [17]. Standardized definitions should be usable across these environments to ensure continuity of care and consistency in clinical practice and research. |
| **11** | Management of febrile discomfort should focus on therapeutic interventions rather than preventive approaches. Research supports treating discomfort based on clinical necessity rather than proactively administering antipyretics without symptoms [18]. Overuse of antipyretics without clear clinical indications is discouraged. |
| **12** | Effective pediatric discomfort management requires regular reassessment following treatment [2]. Clinical guidelines recommend evaluating discomfort within a reasonable timeframe to determine treatment efficacy and adjust care as needed. |

**Note:** **1**. Wensveen FM, Šestan M, Turk Wensveen T, Polić B. 'Beauty and the beast' in infection: How immune-endocrine interactions regulate systemic metabolism in the context of infection. Eur J Immunol 2019; 49:982-995.**2.** Milani GP, Alberti I, Bonetti A, Garattini S, Corsello A, Marchisio P, Chiappini E. Definition and assessment of fever-related discomfort in pediatric literature: a systematic review. Eur J Pediatr 2024; 183:4969-4979.**3**. Doria M, Careddu D, Ceschin F, Libranti M, Pierattelli M, Perelli V, Laterza C, Chieti A, Chiappini E. Understanding Discomfort in Order to Appropriately Treat Fever. Int J Environ Res Public Health 2019; 16.**4**. Ibarra-Coronado EG, Pantaleón-Martínez AM, Velazquéz-Moctezuma J, Prospéro-García O, Méndez-Díaz M, Pérez-Tapia M, Pavón L, Morales-Montor J. The Bidirectional Relationship between Sleep and Immunity against Infections. J Immunol Res 2015; 2015:678164.**5.** Widyadharma IPE, Dewi PR, Wijayanti IAS, Utami DKI. Pain related viral infections: a literature review. Egypt J Neurol Psychiatr Neurosurg 2020; 56:105.**6**. Vandenberk M, De Bondt K, Nuyts E, Toelen J, Verbakel JY. Shivering has little diagnostic value in diagnosing serious bacterial infection in children: a systematic review and meta-analysis. Eur J Pediatr 2021; 180:1033-1042.**7.** Varni JW, Limbers CA, Burwinkle TM. How young can children reliably and validly self-report their health-related quality of life?: an analysis of 8,591 children across age subgroups with the PedsQL 4.0 Generic Core Scales. Health Qual Life Outcomes 2007; 5:1.**8.** Gennis HG, Bucsea O, Badovinac SD, Costa S, McMurtry CM, Flora DB, Pillai Riddell R. Child Distress Expression and Regulation Behaviors: A Systematic Review and Meta-Analysis. Children (Basel) 2022; 9:1749.**9.** Hague R. Managing the child with a fever. Practitioner 2015; 259:17-21, 12-13.**10**. Whalley K. Coordinating sickness behaviour. Nat Rev Neurosci 2022; 23:456-457.**11**. Trottier ED, Ali S, Doré-Bergeron MJ, Chauvin-Kimoff L. Best practices in pain assessment and management for children. Paediatr Child Health 2022; 27:429-448.**12.** Nigrovic LE, Mahajan PV, Blumberg SM, Browne LR, Linakis JG, Ruddy RM, Bennett JE, Rogers AJ, Tzimenatos L, Powell EC, Alpern ER, Casper TC, Ramilo O, Kuppermann N. The Yale Observation Scale Score and the Risk of Serious Bacterial Infections in Febrile Infants. Pediatrics 2017; 140.**13**. Burstein B, Lirette MP, Beck C, Chauvin-Kimoff L, Chan K. Management of well-appearing febrile young infants aged ≤90 days. Paediatr Child Health 2024; 29:50-66.**14.** Chiappini E, Bortone B, Galli L, de Martino M. Guidelines for the symptomatic management of fever in children: systematic review of the literature and quality appraisal with AGREE II. BMJ Open 2017; 7:e015404**.15**. Green R, Webb D, Jeena PM, Wells M, Butt N, Hangoma JM, Moodley RS, Maimin J, Wibbelink M, Mustafa F. Management of acute fever in children: Consensus recommendations for community and primary healthcare providers in sub-Saharan Africa. Afr J Emerg Med 2021; 11:283-296**.16.** Barbi E, Marzuillo P, Neri E, Naviglio S, Krauss BS. Fever in Children: Pearls and Pitfalls. Children (Basel) 2017; 4.**17**. Ogunyinka IA, Oshikoya KA, Yusuff KB, Tahir Y, Yahaya M, Adeniye SB, Oforkansi IE. Determinants of caregiver's knowledge and practices regarding childhood fever management in a developing setting: a multi-centre cross-sectional assessment. Front Pediatr 2023; 11:1119067.**18**. Chiappini E, Venturini E, Remaschi G, Principi N, Longhi R, Tovo PA, Becherucci P, Bonsignori F, Esposito S, Festini F, Galli L, Lucchesi B, Mugelli A, Marseglia GL, de Martino M. 2016 Update of the Italian Pediatric Society Guidelines for Management of Fever in Children. J Pediatr 2017; 180:177-183.e17.

**Detailed description of the results**

All panel experts completed the iterated surveys, which consisted of three rounds. All panel experts completed the iterated surveys, which consisted of three rounds. The results of the first, second, and third rounds are summarized in Table 1. After the first round, a high level of consensus was observed for 5 out of 20 statements. During the second round, seven statements were assessed; in particular, one new tool, the Traffic Light System, was introduced (including one statement on its use), which incorporated 9 of the previous 15 statements that had not reached consensus during the first round, while the remaining 6 of 15 statements were reassessed without any incorporation. Of the seven statements reassessed in the second round, three required a third round to achieve at least 75% agreement, and one statement (Statement #7) did not reach agreement.

All experts agreed that fever-associated discomfort cannot be defined as a single sign or symptom; rather, it comprises multiple clinical indicators that may present concurrently or subsequently (Statement #1, 1^st^ round, 100% agreement).

To adequately assess fever-associated discomfort, the panel was also asked to consider the definition of fever itself. They were initially asked to express their agreement with the World Health Organization (WHO) definition of fever (axillary temperature >37.5°C) as reported in Statement #7 (Supplementary Table S1). However, this definition was not approved by any expert. Higher agreement (71%) was achieved in the second and third rounds by introducing the concept that there is no single temperature cut-off, site of the body for its measurement or single definition of fever to be considered when assessing fever-related discomfort. In the third round, only 71% of experts agreed that **the definition of fever varies in the literature and depends on national guidelines or local protocols. Thus, no final consensus was reached on this statement.**

During the first round, most of the comments focused on identifying the most relevant clinical indicators for defining fever-associated discomfort (Statements #3a–3i). Most proposed indicators did not achieve full agreement. The only indicator that reached high consensus (86%) was moaning or a suffering facial expression. A total of 71% of experts agreed that the following indicators define fever-related discomfort: diffuse myalgia or arthralgia, feeling unwell, prolonged or inconsolable crying, irritability or restlessness, and sleep disturbances (difficulty falling asleep, frequent awakenings, or waking earlier than usual). Only 57% of experts agreed that the following could be considered indicators of fever-related discomfort: reduced ability to maintain adequate hydration and caloric intake over the previous 8 hours, chills or goosebumps, and fatigue or reduced responsiveness (Table 1).

In line with the experts' comments and considering the high disagreement among indicators of fever-related discomfort, during the second round, a “Traffic Light System” was proposed by the steering committee and voted on (and in some cases commented) by the panellists (Figure 1). This system included all indicators of fever-related discomfort from the nine statements voted on in the first round and stratified them into three categories: “green light” for indicators compatible with discomfort that do not require treatment; “amber light” included signs and symptoms compatible with mild to moderate discomfort, for which treatment should be considered on a case-by-case basis; finally “red light” included signs or symptoms that should always be treated. The Traffic Light System did not reach a consensus in the second round and was further modified considering the new comments raised by the panellists. In the third round, all experts agreed on both the signs and symptoms to be considered indicators of fever-related discomfort included in the Traffic Light System and on their stratification, which differentiates between milder signs and those indicating greater severity of fever-related discomfort (Figure 1). Moreover, all experts agreed to use the Traffic Light System for the identification and management of fever-related discomfort (Traffic Light System statement, 3^rd^ round, 100% agreement). The modifications introduced in the third round that led to full consensus were as follows: for the green light, signs/symptoms included do not require treatment unless other factors are present. During the first round, only 71% of experts agreed that if at least one indicator of discomfort is present in a child with fever, discomfort should be diagnosed and managed accordingly. In the second round, even fewer experts (57%) agreed that, if at least one indicator of discomfort is present in a child with fever, discomfort should be considered (Statement #2). In the third round, after the introduction of the Traffic Light System, 86% of experts agreed that if at least one of the indicators (amber and red signs/symptoms in the traffic light system) is present in a child with fever, the presence of a potentially severe discomfort should be considered.

During the Delphi process, 86% of experts agreed that a child’s “well-being” should not be considered synonymous with comfort or discomfort (Statement #5, 2^nd^ round). The panel emphasized that discomfort is distinct from global well-being, as fever-related discomfort reflects acute, symptom-driven clinical distress rather than broader psychological or psychosocial domains captured by well-being instruments.

Experts agreed that pain may be a component of discomfort, but that these two concepts are not synonymous. Indeed, all experts agreed that localized pain (e.g., otalgia, pharyngodynia) should be assessed and managed using validated pain scales. However, this did not preclude localized pain from being considered a sign or symptom of fever-associated discomfort (Statement #4, 2^nd^ round; agreement 100%).

The definition of discomfort was considered applicable across the pediatric age spectrum, including preverbal children, children aged 4–8 years, and those aged 8-17 years (Statement #6, 2^nd^ round, 86% agreement). However, experts acknowledged that the definition of fever-associated discomfort cannot be universally applied to all children, as underlying medical conditions or care settings may influence clinical signs and symptoms. Therefore, in the third round, experts agreed that the applicability of this definition should be considered on a case-by-case basis for children with neurological conditions, heart disease, immunological disorders, or receiving immunosuppressive therapy; those with severe metabolic disorders or severe lung diseases (e.g., bronchopulmonary dysplasia); those with homozygous sickle cell disease; and those admitted to intensive care units (Statement #8, 86% agreement).

The panel also agreed that the definition of fever-associated discomfort is not intended solely for physicians. Specifically, 86% of experts agreed that the definition can be applied by nurses and other healthcare professionals involved in routine pediatric care (Statement #9, 1^st^ round). Furthermore, the definition is not restricted to hospital use and can be applied in outpatient clinics, inpatient settings, and community environments, such as home or school (Statement #10, 1^st^ round, 86% agreement).

Regarding therapeutic strategies, 86% of experts agreed that the management of febrile children with discomfort should focus exclusively on therapeutic interventions rather than preventive measures (Statement #11, 86% agreement). Moreover, 86% agreed that after administering treatment for discomfort, it is appropriate to reassess the child’s condition after a reasonable period (Statement #12, 86% agreement).
